# Influence of Primary Care Physicians on End-of-Life Treatment Choices in Lung Cancer Diagnosed in the Emergency Department

**DOI:** 10.3390/jpm15080339

**Published:** 2025-08-01

**Authors:** Tatsuyuki Kawahara, Nobuaki Ochi, Hirohito Kirishi, Yusuke Sunada, Ayaka Mimura, Naruhiko Ichiyama, Yoko Kosaka, Yasunari Nagasaki, Hidekazu Nakanishi, Hiromichi Yamane, Nagio Takigawa

**Affiliations:** Department of General Internal Medicine 4, Kawasaki Medical School, Okayama 701-0192, Japan; kawatatsu.0622@gmail.com (T.K.); ntakigaw@med.kawasaki-m.ac.jp (N.T.)

**Keywords:** lung cancer, emergency department, primary care physician, shared decision making, end-of-life

## Abstract

**Background**: Lung cancer remains one of the leading causes of cancer-related mortality worldwide. While most diagnoses occur in outpatient settings, a subset of cases are incidentally identified during emergency department (ED) visits. The clinical characteristics and treatment decisions of these patients, particularly in relation to social background factors such as living situation and access to primary care, remain poorly understood. **Methods**: We conducted a retrospective study of patients diagnosed with malignancies in the ED of a single institution between April 2018 and December 2021. Patients diagnosed with lung cancer within 60 days of an ED visit were included. Data on demographics, disease status, treatment decisions, and background factors—including whether patients lived alone or had a primary care physician (PCP)—were extracted and analyzed. **Results**: Among 32,108 patients who visited the ED, 148 were diagnosed with malignancy within 60 days; 23 had lung cancer. Of these, 69.6% had metastatic disease at diagnosis, and 60.9% received active treatment (surgery or chemotherapy). No significant associations were observed between the extent of disease and either living arrangement or PCP status. However, the presence of a PCP was significantly associated with the selection of best supportive care (*p* = 0.023). No significant difference in treatment decisions was observed based on age (cutoff: 75 years). **Conclusions**: Although social background factors such as living alone were not significantly associated with cancer stage or treatment choice, the presence of a primary care physician was associated with a higher likelihood of best supportive care being selected. This may indicate that patients with an established PCP have more clearly defined care goals at the end of life. These findings suggest that primary care access may play a role in shaping end-of-life care preferences, highlighting the importance of personalized approaches in acute oncology care.

## 1. Introduction

Lung cancer remains one of the leading causes of cancer-related mortality worldwide, accounting for a significant proportion of cancer deaths annually [1,2]. Early detection and appropriate therapeutic strategies are critical for improving survival outcomes in patients with lung cancer [3,4]. The route to lung cancer diagnosis varies widely, and increasing attention has been paid to patients diagnosed through emergency presentations. However, reports on patients with malignancies presenting to the emergency department remain limited [5,6,7]. Jane et al. reported that approximately one-third of such patients had hematologic malignancies in a retrospective study. Other common malignancies included breast (14%), gastrointestinal (13%), and lung cancer (11%) [5]. When patients present to the emergency department (ED), they may exhibit a wide range of symptoms, from mild to severe. Common symptoms include chest pain, shortness of breath, abdominal pain, fever, and neurological changes. Patients may present with a single symptom or a combination of symptoms, and the severity can vary greatly. It has been reported that patients diagnosed with lung cancer in the ED generally have poorer prognoses, often presenting with advanced-stage disease and limited eligibility for curative treatment. In one study, more than one-third of such patients died within 90 days, and only a minority received systemic anti-cancer therapy [8]. In the United Kingdom, data from national audits have shown that 19–39% of lung cancer cases are diagnosed via emergency presentations, and these patients have markedly lower 1-year survival compared to those diagnosed electively [7]. In the United States, a large SEER-Medicare study reported that nearly 40% of lung cancer diagnoses involved an ED visit in the preceding month [9]. These findings reflect systemic and access-related differences in cancer detection across countries.

It is important for healthcare providers in the ED to promptly assess a patient’s symptoms and medical history in order to provide timely and appropriate care. In many countries, including Japan, primary care physicians (PCPs) play a crucial role in promoting daily health and managing chronic conditions, such as lifestyle-related diseases. Patients with a regular PCP may have greater health awareness and an increased likelihood of early cancer detection, whereas those without one may miss such opportunities. For instance, a recent U.S. study revealed that only 16% of patients diagnosed with lung cancer in the ED had a regular primary care provider, and among those eligible for screening, merely 6% had been screened prior to diagnosis [6]. This suggests that the absence of regular primary care may contribute to delayed or incidental cancer detection. Meanwhile, some patients are incidentally diagnosed with malignant tumors during emergency room visits. There is no comprehensive report on such cases. However, the relationship between patient background factors—such as living situation and primary care physician involvement—and both prognosis and treatment decisions in incidentally diagnosed lung cancer remains poorly understood. Therefore, in this study, we assessed the frequency of lung cancer cases incidentally diagnosed during emergency room visits and examined the association between their background factors (such as living situation and the presence of a primary care physician), the extent of disease at diagnosis, and treatment selection. Understanding these factors may contribute to the implementation of more personalized and patient-centered care strategies in emergency settings.

## 2. Methods

### 2.1. Patient Selection and Data Extraction

This retrospective study was conducted using electronic medical record data from consecutive patients diagnosed with malignancy in the ED of a single institution, Kawasaki Medical School, Kawasaki Medical Center, between April 2018 and December 2021. Our hospital serves both as a university-affiliated and community-based facility in a regional urban area.

In this study, the term “diagnosed malignancy” was operationally defined as a new cancer diagnosis confirmed within 60 days of the ED visit, based on either (1) pathological confirmation (e.g., biopsy or cytology), or (2) radiographic findings highly suggestive of malignancy supported by clinical or laboratory data (e.g., elevated tumor markers or physician documentation).

We chose a 60-day cutoff to capture cases with a meaningful temporal relationship to the ED encounter. Although no standardized timeframe has been established in prior literature, we judged that 30 days might be insufficient to complete the full diagnostic process, particularly for patients with complex social backgrounds or delayed follow-up. A 60-day window was considered more practical for allowing diagnostic work-up, pathological confirmation, and treatment planning, while still reflecting cases related to the ED visit.

The inclusion criteria were as follows:

(1) New diagnosis of malignancy within 60 days of the index ED visit;
(2) Diagnosis made at our hospital;
(3) Age ≥18 years.

The exclusion criteria were as follows:

(1) Patients referred from other institutions with prior suspicion or known diagnosis of malignancy;
(2) Patients with a previously documented cancer diagnosis before the ED visit;
(3) Non-solid tumors (e.g., leukemia) or benign neoplasms.

This study was approved by the Ethics Committee of Kawasaki Medical School (approval number: 5147, approval date: 3 February 2021). Data collection commenced after approval and continued prospectively through the end of the study period. The dataset included information on patient demographics, cancer type, histology, ECOG performance status (PS), smoking history, treatment selection, and social background factors, such as living situation and PCP status.

### 2.2. Statistical Analysis

Associations between patient background factors and either the extent of cancer progression at diagnosis or the selected treatment modality were assessed using 2 × 2 contingency tables. Statistical significance was evaluated using the chi-square test or Fisher’s exact test, as appropriate. A *p*-value of <0.05 was considered statistically significant. All statistical analyses were performed using Stata software (version 18, StataCorp), ensuring a robust and comprehensive evaluation of study outcomes. To minimize bias in data abstraction, two physicians (NO and TK; NO is board-certified in medical oncology) independently extracted data from the electronic medical records and compared their results to ensure consistency.

## 3. Results

**Figure 1:**
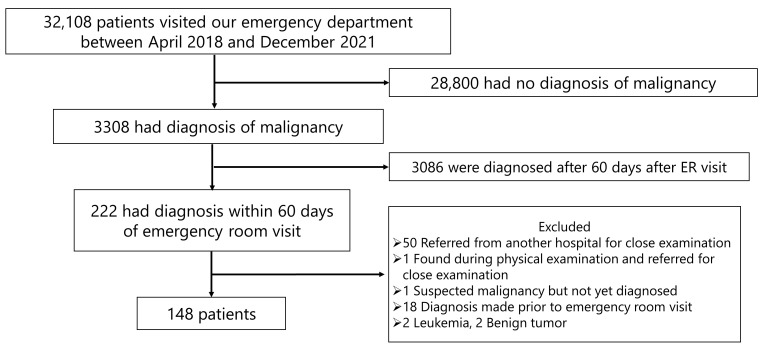
Flowchart illustrating patient selection in this study.

Figure 1 shows the patient selection flow for this study. From April 2018 to December 2021, a total of 32,108 patients visited the ED of our hospital. Of these, 28,800 patients were excluded because they had no diagnosis of malignancy. Additionally, 3086 patients were diagnosed with malignancy more than 60 days after their ED visit and were also excluded. Among the remaining 222 patients, further exclusions were made for the following reasons: 50 patients were referred from other hospitals for close examination; one was referred after a physical examination at our hospital; one had suspected malignancy but was not yet diagnosed; 18 had already been diagnosed with malignancy prior to their ED visit; and 4 had non-solid tumors (2 leukemia and 2 benign tumors). After these exclusions, a total of 148 patients were included in the final analysis.

The distribution of primary tumor types among the 148 included patients is summarized in Table 1. The most common was gastrointestinal cancer (33.1%), followed by hepatobiliary and pancreatic cancer (15.5%), lung cancer (15.5%), urologic cancer (10.1%), lymphoma (6.8%), breast cancer (5.4%), gynecologic cancer (3.4%), and others (10.8%). Table 2 shows the histological subtypes of the 23 lung cancer cases. Adenocarcinoma was the most frequent subtype (14 cases, 60.8%), followed by squamous cell carcinoma (4 cases, 17.4%), small cell carcinoma (3 cases, 13.0%), and others (2 cases, 8.6%).

Patient characteristics are summarized in Table 3. The median age was 75 years (range, 53–96 years), and males accounted for a slightly higher proportion (60.9%) than females. A relatively high number of patients (73.9%) had a good PS (0–1). Among patients with known smoking status, 65.2% were smokers, and the majority of smokers (86.7%) had a Brinkman index of 400 or higher, suggesting a history of heavy smoking. At the time of diagnosis, 69.6% of patients had metastatic disease, and 21.7% had multiple cancers. Regarding social background, 43.5% of patients lived alone, and 65.2% reported having a regular PCP. Next, we assessed the association between patient background factors and the extent of cancer progression at diagnosis (Table 4). Among patients living alone, 80% had metastatic disease at the time of diagnosis, compared to 61.5% of those living with family. However, this difference was not statistically significant (*p* = 0.405, Fisher’s exact test). Similarly, 87.5% of patients without a PCP had metastatic disease, compared to 60% of those with one. Again, the difference did not reach statistical significance (*p* = 0.345). These findings suggest a possible trend, but no clear association between living arrangement or primary care access and cancer stage at diagnosis was identified.

We then analyzed the relationship between treatment choice and patient background factors (Table 5). Of the 23 patients with lung cancer, one case in which the treatment decision was not documented was excluded from this analysis; among the remaining 22 patients, surgery was chosen in 4 cases and chemotherapy in 11 cases. Of the remaining 8 patients, 7 received best supportive care (BSC), and 1 case was classified as unknown. In terms of living situation, 80% of patients living alone received active treatment, while 61.5% of those receiving BSC also lived alone. However, this difference was not statistically significant (*p* = 1.000). In contrast, the presence of a PCP showed a significant association with treatment choice (*p* = 0.023). All patients who received BSC had a PCP, while among those who received active treatment, 46.7% had a PCP and 53.3% did not. We also examined whether age influenced treatment selection using a cutoff of 75 years, but no statistically significant difference was found.

## 4. Discussion

This study aimed to investigate the characteristics and treatment decisions of patients incidentally diagnosed with lung cancer following an ED visit. It is not uncommon for patients with malignant tumors to be incidentally diagnosed following an ED visit, as observed in our study (0.46%, 148/32,108 cases). Among these, lung cancer ranked third, with a total of 23 cases (14%). Although the number of cases was relatively small, previous studies have highlighted the important role of the ED in the initial diagnosis of cancer. A recent scoping review reported that cancers diagnosed in the ED are often incidental or present acutely, and such cases tend to involve patients with more comorbidities, advanced-stage disease, and poorer prognoses. These findings suggest that the ED may serve as a critical—yet underrecognized—entry point in the cancer care continuum [7]. An analysis of nationwide ED data in the United States revealed that 4.2% of cancers were diagnosed in the ED [6]. The most common malignancies associated with ED presentation were breast, prostate, and lung cancers, with the most frequent presenting symptoms including pneumonia (4.5%), nonspecific chest pain (3.7%), and urinary tract infection (3.2%). Compared to our findings, the proportion reported in that study was considerably higher. This discrepancy may reflect differences in study populations, healthcare system accessibility, and the influence of Japan’s universal healthcare system, which may lower psychological and financial barriers to seeking emergency care. In a retrospective study of 771 patients with advanced NSCLC, Fujimoto et al. reported that 13% were diagnosed following emergency admission (DFEA). The median overall survival was significantly shorter in the DFEA group (6.4 months) compared to the non-DFEA group (16.6 months). However, multivariate analysis demonstrated that DFEA itself was not an independent prognostic factor. Instead, PS at the start of chemotherapy was the strongest predictor of overall survival (hazard ratio 0.26; *p* < 0.001). These findings emphasize the clinical importance of improving PS after admission rather than focusing solely on the diagnostic route [10].

Treatment decision-making in cancer care is influenced by various factors, including age, PS, comorbidities, cognitive function, activities of daily living (ADL), disease stage, financial concerns, and the presence of supportive caregivers or family members. In elderly patients, emotional and physical support from family or caregivers is particularly important when considering aggressive treatments such as surgery or chemotherapy [11]. For example, a study in postmenopausal women with colorectal cancer showed that living alone was not associated with mortality in the overall cohort, but both social integration and living alone were linked to poorer outcomes in patients with rectal cancer [12]. Similarly, a population-based study involving over 260,000 patients with late-stage cancer in the United States found that unmarried individuals—particularly those who were single or widowed—had significantly worse survival outcomes than their married counterparts, even after adjusting for confounding variables. This trend was especially pronounced in men [13]. Although the exact mechanisms remain unclear, these findings suggest that social isolation and a lack of support may negatively influence cancer prognosis.

In our study, we initially hypothesized that patients living alone might delay seeking medical attention when symptoms appeared, and that patients without a PCP might lack routine medical follow-up, leading to diagnosis at more advanced stages. However, no significant association was found between either living situation or PCP status and the presence of metastasis at diagnosis. This may be partially attributable to the limited sample size. On the other hand, the presence of a PCP was significantly associated with the selection of BSC. Although the underlying reasons remain unclear, it is plausible that PCPs, who have a deeper understanding of patients’ clinical and social backgrounds, may have guided them toward appropriate care goals, including palliative or non-curative approaches. Conversely, 60% of patients received active treatment, such as surgery or chemotherapy, underscoring the critical importance of emergency physicians conducting timely and appropriate evaluations—including patient history, PS, and physical examination—while remaining vigilant for the possibility of underlying malignancies. Additionally, no significant difference in treatment selection was observed based on age when using 75 years as the cutoff, suggesting that age alone may not be the determining factor in therapeutic decision-making in this cohort.

In addition to lung cancer, other major malignancies, such as gastrointestinal, hepatobiliary, breast, and hematologic cancers, are also frequently diagnosed in the emergency department. In our cohort, gastrointestinal cancers were the most common, accounting for 33% of all ED-diagnosed cases. This is consistent with prior studies indicating that colorectal and hepatobiliary cancers often present emergently due to symptoms such as obstruction, bleeding, or jaundice. A population-based study showed that 39% of colorectal cancer patients were diagnosed via emergency presentation, which was associated with worse survival outcomes compared to elective diagnosis [9]. Breast cancer, in contrast, is less frequently diagnosed in emergency settings due to the effectiveness of screening programs, although cases presenting acutely often reflect advanced-stage or neglected disease. Hematologic malignancies—especially aggressive lymphomas and acute leukemias—are also commonly diagnosed in the ED, often with acute symptoms like cytopenia, organ compression, or infection. One retrospective study reported that hematologic cancers comprised up to one-third of ED-diagnosed cancers [7]. These findings highlight the diverse oncologic presentations encountered in emergency settings and underscore the importance of prompt recognition and interdisciplinary collaboration in managing such patients. They also suggest that insights gained from lung cancer cases may have broader relevance across malignancies.

Furthermore, our findings may have implications for shared decision-making (SDM) and advance care planning (ACP), particularly in the context of emergency care. In Japan, the concept of SDM is evolving, with increasing emphasis on aligning treatment decisions with patient values and care goals. Patients with an established primary care physician may have had prior discussions regarding prognosis and treatment preferences, which could explain the higher rate of best supportive care in this group. Studies from palliative care settings suggest that integrating ACP into routine care facilitates timely, patient-centered decisions, especially when health deteriorates rapidly [11,14]. From a global perspective, the role of primary care varies across countries. In nations like the UK, where primary care serves as a formal gatekeeper to specialist care, early diagnosis may be more systematic, while in Japan or China, patients often access tertiary care directly without a referral [15,16,17]. This difference may partially explain the variations in emergency presentations and stage at diagnosis. Strengthening primary care engagement, including ACP and SDM, could be a key strategy to optimize cancer care pathways and reduce the burden of emergency diagnoses [15,17].

There are several limitations to this study. First, the overall sample size was small, as patients diagnosed with malignancy within 60 days of an ER visit accounted for less than 0.5% of all ER visits. Lung cancer patients represented only 0.07% of the total study population. Second, this was a retrospective study based on electronic medical record review. Although the data were carefully collected, some information may have been incomplete or unavailable. Particularly, history-taking and initial diagnostic impressions may have been influenced by the experience and clinical judgment of the attending emergency physicians. Third, the actual number of malignancy cases may have been underestimated. Patients with severe symptoms were more likely to undergo detailed investigations such as CT or MRI, increasing the likelihood of malignancy detection. Conversely, those with mild symptoms may have harbored undiagnosed cancers that went unnoticed due to limited diagnostic work-up at the time of the ED visit. Therefore, the findings should be interpreted with caution. The small sample size and single-center, retrospective design limit the generalizability of our results and may increase the potential for selection and interpretation bias. These limitations underscore the need for further validation in larger and more diverse populations. Further prospective, multicenter studies with larger sample sizes are warranted to better clarify the influence of patient background factors, including social support and primary care access, on cancer diagnosis and treatment decisions in emergency settings.

Although the data used in this study were collected between 2018 and 2021, the essential role of primary care physicians as the most accessible and longitudinal healthcare providers has remained consistent. While there may have been some evolution in primary care models and healthcare policies in recent years, we believe that our findings continue to hold relevance, particularly in the context of shared decision-making (SDM), which has gained even greater emphasis in contemporary oncology practice. Future research will be valuable in evaluating how these roles evolve and how they may further enhance personalized, end-of-life care in emergency settings.

Given the limited sample size and retrospective nature of this study, future prospective, multi-institutional research is warranted to validate our findings. In particular, studies exploring the role of primary care engagement and its influence on shared decision-making, quality of life, and end-of-life care in larger lung cancer populations would be valuable. Additionally, qualitative studies incorporating patient and caregiver perspectives may offer deeper insights into the decision-making process in emergency settings.

## 5. Conclusions

In conclusion, our findings suggest that social and healthcare support systems, particularly the presence of a PCP, may play a role in shaping treatment decisions in patients diagnosed with lung cancer during emergency visits. As cancer care increasingly emphasizes personalized and context-aware approaches, understanding these background factors may be key to ensuring timely, appropriate, and patient-centered interventions. These results support the incorporation of individual patient backgrounds and pre-existing care relationships into therapeutic decision-making, in line with the principles of personalized medicine.

## Figures and Tables

**Table 1:** Distribution of primary tumor types among included patients (n = 148).

Tumor Type	No. (%)
Gastrointestinal	49, (33.1%)
Hepatobiliary/Pancreatic	23, (15.5%)
Lung	23, (15.5%)
Urologic	15, (10.1%)
Lymphoma	10, (6.8%)
Breast	8, (5.4%)
Gynecologic	5, (3.4%)
Others	16, (10.8%)

**Table 2:** Histological subtypes of lung cancer cases (n = 23).

Histological Subtype	No. (%)
Adenocarcinoma	14, (60.8%)
Squamous cell carcinoma	4, (17.4%)
Small cell carcinoma	3, (13.0%)
Others	2, (8.6%)

**Table 3:** Patient characteristics of the 23 lung cancer cases diagnosed in the emergency department.

Variables	No. (%)
Median age (range)—year	75.0 (53–96)
Gender	
Male/Female	14 (60.9)/9 (39.1)
Performance status	
<2/≥2	17 (73.9)/6 (26.1)
Smoker	
Yes/No	15 (65.2)/7 (30.4)
Unknown	1 (4.3)
Smoking status	
20 PY/≥20 PY	1 (6.7)/13 (86.7)
Unknown	1 (6.7)
Metastatic disease	
Yes/No	16 (69.6)/7 (30.4)
Multiple cancers	
Yes/No	5 (21.7)/18 (78.3)
Living alone	
Yes/No	10 (43.5)/13 (56.5)
Presence of PCP	
Yes/No	15 (65.2)/8 (34.8)

Abbreviations: PY; pack per year, and PCP; primary care physician.

**Table 4:** Association between patient background factors and the extent of cancer progression at diagnosis.

	Metastasis		
Variables	Yes	No	*p*-Value
Living situation			
Lives alone	8, (80.0%)	2, (20.0%)	
Lives with family	8, (61.5%)	5, (38.5%)	*p* = 0.405
Primary care physician			
Yes	9, (60.0%)	6, (40.0%)	
No	7, (87.5%)	1, (12.5%)	*p* = 0.345

**Table 5:** Association between patients’ treatment choice and the presence of a primary care physician.

	Treatment Choice		
Variables	Any Treatment	BSC	*p*-Value
Living situation			
Lives alone	7, (80.0%)	3, (61.5%)	
Lives with family	8, (20.0%)	4, (38.5%)	*p* = 1.000
Primary care physician			
Yes	7, (46.7%)	7, (100%)	
No	8, (53.3%)	0, (0.0%)	*p* = 0.023

Abbreviations: BSC; best supportive care.

## Data Availability

The datasets used and/or analyzed during the current study are available from the corresponding author on reasonable request.
